# Antenatal Diagnosis of a Partial Atrioventricular Canal with Ebstein’s Anomaly

**DOI:** 10.3390/children8111029

**Published:** 2021-11-10

**Authors:** Gerald Laforest, Jean-Bernard Selly, Gilbert Dubois, Bernard Kreitmann, Yael Levy

**Affiliations:** 1 Department of Pediatric Cardiology, University Hospital, 97400 Saint-Denis, France; gerald.laforest@chu-reunion.fr (G.L.); jean-bernard.selly@chu-reunion.fr (J.-B.S.); 2 Department of Cardiac Surgery, University Hospital, 97400 Saint-Denis, France; gilbert.dubois@chu-reunion.fr; 3 Cardiac Surgery Unit, Bordeaux University Hospital, 33000 Bordeaux, France; bernard.kreitmann@u-bordeaux.fr; 4 Department of Pediatric Cardiac Intensive Care, University Hospital, 97400 Saint-Denis, France

**Keywords:** atrioventricular canal, Ebstein’s anomaly, antenatal diagnosis

## Abstract

The simultaneous occurrence of an atrioventricular canal defect (AVCD) and Ebstein’s anomaly is extremely rare, occurring in less than 0.5% of all patients with AVCD. Only 22 cases are described in the literature. This patient’s antenatal diagnosis of both Ebstein’s anomaly and partial AVCD was made at 25 weeks of gestation. The delivery was organized in a tertiary center. The initial neonatal course was difficult but with adequate treatment, a rapid improvement allowed for a gap of almost 2 years before a complete surgical repair including a cone tricuspid plasty. To our knowledge, this is the first case of antenatal diagnosis, with carefully tailored delivery, neonatal care and subsequent follow-up before indication for successful surgery.

## 1. Introduction

Ebstein’s anomaly (EA) is a rare congenital heart disease (CHD) representing 1 to 5 per 200,000 live births, accounting for less than 1% of CHD cases [1]. Atrioventricular canal defects (AVCD) are more prevalent and are associated with a wide variety of congenital cardiac malformations. However, the simultaneous occurrence of AVCDs and EA is extremely rare, evaluated to occur in 0.3% of patients with an AVCD [2]. We performed a literature review and found 22 cases, including 16 partial, 3 intermediate and 3 complete AVCD cases. In most cases, the diagnosis was made postmortem via necropsy or during surgery [1,3,4]. The ages of the surgically managed patients ranged from 0.25 to 20 years. Among the 10 patients operated upon, 4 deaths were noted. Herein, we reported the first antenatal documented case of EA with a partial AVCD to our knowledge.

## 2. Case Presentation

**Figure 1:**
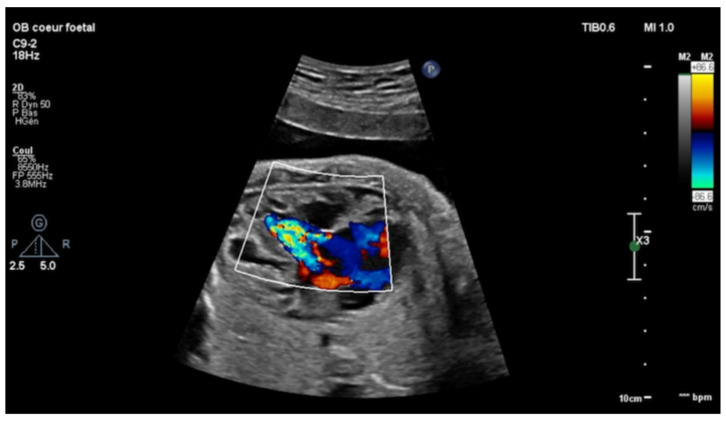
Four-chambers view: color Doppler: tricuspid insufficiency from the apex due to Ebstein’s anomaly.

**Figure 2:**
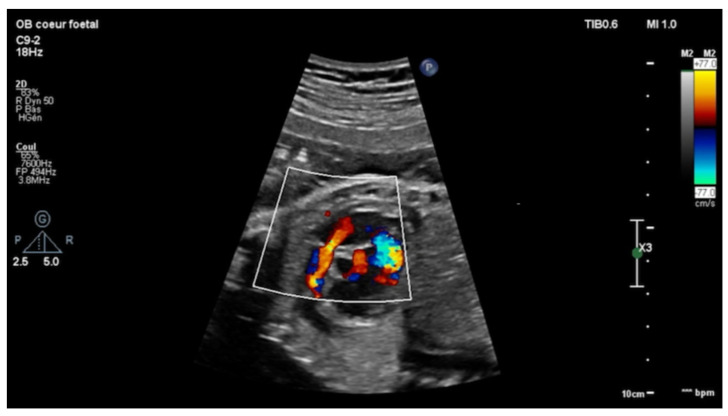
Short-axis view: color Doppler: retrograde flow in the pulmonary artery with pulmonary insufficiency.

Echocardiography in a fetus at 25 weeks of gestation (WG) from a 19-year-old woman, without history of familial CHD, showed: (i) a typical partial common AVCD with a cleft on the left atrioventricular valve (AVV) without stenosis or leak and a large ostium primum atrial septal defect (ASD); (ii) Ebstein abnormality of the right AVV with a significant leak, causing a dilatation of the right atrium (RA) (Figure 1; Videos S1 and S2); (iii) “functional pulmonary atresia” with inconstant anterograde passage and (iv) retrograde flow in a tortuous ductus arteriosus. Other cardiac structures were normal. Cytogenetic analysis via amniocentesis had normal results. At 30 WG, moderate atrialization of the right ventricle and a pulmonary insufficiency were noted (Figure 2).

After vaginal birth at 37 WG, she was polypneic and cyanosed, with a pulse oxymetry at 70%. There was a cardiomegaly on the chest X-ray. The EKG showed no pre-excitation syndrome and a typical left QRS axis. The postnatal transthoracic echocardiography (TTE) confirmed the diagnosis. The right ventricle was functional with an insufficient antegrade pulmonary flow, so-called “functional pulmonary atresia”. Color Doppler imaging showed severe regurgitation of the right AVV and right to-left shunt through the ostium primum defect, but the left-sided AVV was not restrictive and only leaked minimally. The Great Ormond Street Echo (GOSE) score was 0.7 (grade 2). Nevertheless, the large partial AVSD was an obstacle in calculating the GOSE score accurately. The initial management consisted of alprostadil infusion, nitric oxide (NO) and non-invasive ventilation. A specific delivery device of inhaled NO was used with non-invasive ventilation. This device provided a reliable and precise nitric oxide blend with a constant concentration proportional to the ventilatory flow. Indeed, elevated pulmonary vascular resistance may reduce flow across the pulmonary valve. The administration of inhaled NO may help in this situation. During the first 2 weeks, the saturation improved, and treatments were weaned. She was discharged and had a normal somatic growth without treatment. Close follow-up mainly showed the progressive dilation of the right cardiac structures (RA + atrialized right ventricle), and elective surgery was planned at 20 months of age and 10 kg of weight.

**Figure 3:**
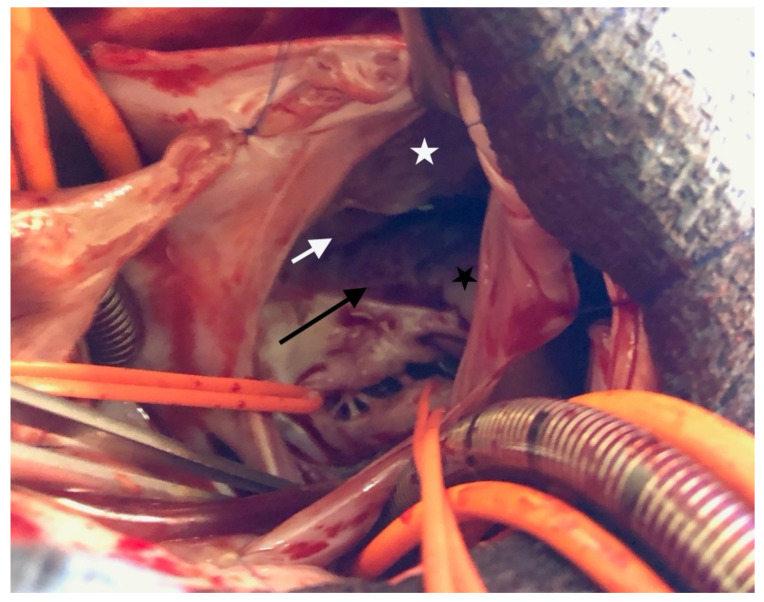
Ebstein’s anomaly. White asterisk: delaminated portion of the anterior valve; black asterisk: posterior valve residue, white arrow: “tricuspid funnel” type opening; black arrow: atrialized portion of right ventricle.

The surgical inspection confirmed the lesions: Ebstein’s disease was atypical and severe and was an intermediate type between B and C [5]. The following description starts from the anteroseptal commissure and goes clockwise: the anteroseptal commissure was very unusual, as if a first part of the anterior valve was missing. A portion of the septal band, the posterior division of the moderator band, reached the atrioventricular valve ring. Then, a small part of the next anterior valve was normally developed. The opening was like the “tricuspid funnel” type and therefore was quite primitively directed towards the infundibulum (Figure 3). Moving to the right and downwards, we quickly found a muscularized valve; then, in some places, a portion was indissociable from the right ventricle (which corresponds more or less to the beginning of the posterior valve, which nevertheless existed), but also, in some other places, was delaminated. Thus, it was unquestionable that it was an Ebstein, although rather atypical. For example, the only real “atrialized” part was in fact at the level of the anterior septal commissure.

**Figure 4:**
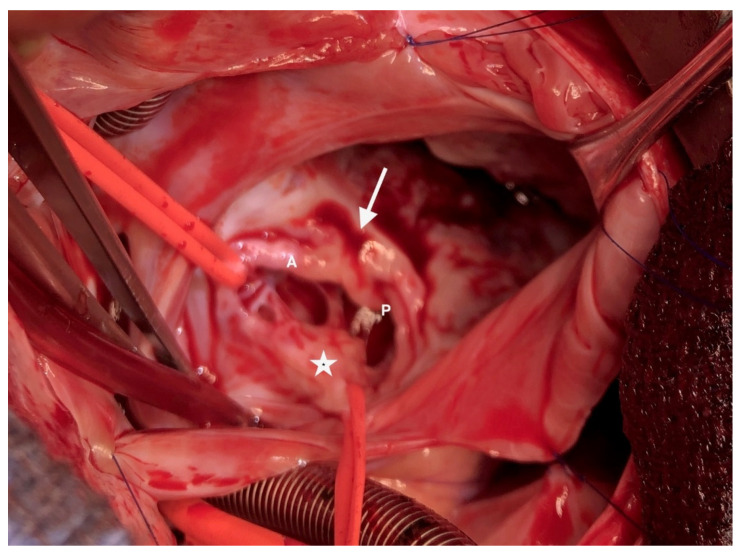
The left atrioventricular valve is composed of 3 leaflets. A: anterior bridging leaflet; P: posterior bridging leaflet; arrow: incomplete cleft; asterisk: left mural leaflet.

Partial AVCD was also indisputable with respect to the left side. There was no ventricular septal defect, and the left atrioventricular annulus was complete and glued to the septal crest. There were three components: one anterior, one posterior and one mural valve (Figure 4). The cleft was not complete; a few mm of tissue united the anterior and posterior components paraseptally. There was a typical ostium primum. Thus, the procedure consisted of a tricuspid “cone” repair with the complete closure of the ostium primum ASD via an autologous pericardial patch and the closure of the left atrioventricular cleft.

Transesophageal echocardiography and TTE showed trivial to mild insufficiency of the left and right AVV, without stenosis, no residual shunt, a moderate dysfunction of the RV free wall and a normal left ventricular function. No rhythm or conduction disorders were noted. The post-operative course was marked by some RV dysfunction (improved with NO and sildenafil). All treatments were rapidly weaned, and a successful extubation was achieved 9 h after her admission to the pediatric cardiac intensive care unit. She was discharged home after 14 days.

One year later, the child is NYHA I, with normal growth. TTE shows a good biventricular function, mild tricuspid insufficiency without stenosis and no stenosis or leak on the left AVV.

## 3. Discussion

To our knowledge, in this study, we reported the first antenatal documented case of Ebstein’s anomaly with a partial AVSD.

The simultaneous occurrence of AVSDs and Ebstein’s anomaly is extremely rare, evaluated to occur in 0.3% of patients with AVSDs. Here, the antenatal diagnosis allowed us to prevent the occurrence of the complications expected in the immediate postnatal period (i.e., increasing resistance vascular pulmonary and inhibition of pulmonary flow). Indeed, the initial management consisted of inhaled NO and prostaglandins infusion to increase pulmonary blood flow. Then, these treatments were weaned due to an improvement in arterial oxygen saturation. This strategy has been reported by Bove et al. [6], who suggest starting prostaglandin and evaluating the presence or absence of circular shunt. If prostaglandin failure to wean and cyanosis is persistent, a systemic to pulmonary artery shunt may be discussed, but if congestive heart failure appears, a closure of the tricuspid valve annulus can be performed with a systemic to pulmonary artery shunt. Tricuspid valve repair or replacement may be discussed in neonates who continue to decline in spite of standard resuscitative measures. Several surgical managements have been reported in the literature [7,8,9]. Among these studies, a large cohort of 23 symptomatic neonates underwent biventricular repair for Ebstein anomaly with an early survival rate of 69.5% [10]. For the neonates without pulmonary atresia, the early survival rate was higher. In this cohort, the GOSE score was greater than 1.5 in 22 of 23 neonates.

We performed a literature review and found 22 documented cases of Ebstein’s anomaly with an AVCD [2,3,4]. The ages of the surgically managed patients ranged from 0.25 to 20 years. Among the 10 patients operated upon, 4 deaths were noted.

The complexity of this association and the lack of documented experience led us to discuss the case in several multidisciplinary meetings. Different pitfalls were possible immediately after birth. The augmentation of systemic afterload could worsen left AVV regurgitation and lead to large left to-right atrial shunting, which could have been poorly tolerated by the right structures. Temporary high post-natal pulmonary resistances, on the contrary, are a well-known risk factor for critical situations in Ebstein disease, and the coexistence of an ostium primum ASD could lead to a very large right to-left shunt with low pulmonary blood flow. The patency of the ductus arteriosus and documented pulmonary regurgitation are the basis of circular shunting [11]. The neonatal management of Ebstein’s anomaly is difficult because the elevated pulmonary vascular resistance of the newborn may inhibit flow across the pulmonary valve, resulting in uncertainty as to whether the outflow tract obstruction is functional or anatomic. In the neonatal period, cyanosis may be reduced by increasing the pulmonary blood flow through the use of prostaglandins. If the pulmonary outflow tract obstruction is functional, a rapid weaning of prostaglandin is indicated after the pulmonary vascular resistance falls.

This led us to prone prenatal transfer and delivery in a tertiary care center. There, immediate treatment aimed toward treatment of the observed consequences (mainly deep cyanosis) and probably prevented more severe decompensation. Indeed, the postnatal management of this case follows the current line reported by several centers with a lot of experience that has improved the survival results [6,12]. After this period, we were able to closely follow-up the evolution of the defects and indicate surgery above one year of age and at the weight of 10 kg, presumably facilitating the obtention of a very good result.

## 4. Conclusions

We reported the first antenatal documented case of Ebstein anomaly with a partial AVCD. The postnatal evolution was unpredictable, but the antenatal diagnosis and multidisciplinary meetings allowed for the optimization of the management, probably contributing to the good surgical result. With these findings, we would like to recommend prenatal transfer and delivery in a tertiary care center in a similar case and would be very interested to hear about other comparable situations.

## Supplementary Materials

The following are available online at https://www.mdpi.com/article/10.3390/children8111029/s1, Video S1: four-chambers view: left: ostium primum and aligned atrioventricular valves. Right: color Doppler: tricuspid insufficiency from the apex due to Ebstrein’s anomaly. Video S2: short-axis view: left: no delaminated septal leaflet (top) and mitral cleft (bottom). Right: color Doppler: tricuspid insufficiency (top), absence of mitral insufficiency on the cleft (bottom).
